# FAD binding, cobinamide binding and active site communication in the corrin reductase (CobR)

**DOI:** 10.1042/BSR20140060

**Published:** 2014-07-04

**Authors:** Andrew D. Lawrence, Samantha L. Taylor, Alan Scott, Michelle L. Rowe, Christopher M. Johnson, Stephen E. J. Rigby, Michael A. Geeves, Richard W. Pickersgill, Mark J. Howard, Martin J. Warren

**Affiliations:** *School of Biosciences, University of Kent, Canterbury, Kent CT2 7NJ, U.K.; †Medical Research Council Laboratory for Molecular Biology, Hills Road, Cambridge CB2 0QH, U.K.; ‡Manchester Institute of Biotechnology, University of Manchester, 131 Princess Street, Manchester M1 7DN, U.K.; §School of Biological and Chemical Sciences, Queen Mary, University of London, Mile End Road, London E1 4NS, U.K.

**Keywords:** enzyme kinetics, enzyme structure, NMR, vitamins and cofactors, X-ray crystallography, CobR, corrin reductase, DSC, differential scanning calorimetry, FAD, flavin dinucleotide, FMN, flavin mononucleotide, HSQC, heteronuclear single-quantum coherence, NOE, nuclear Overhauser effect

## Abstract

Adenosylcobalamin, the coenzyme form of vitamin B_12_, is one Nature's most complex coenzyme whose *de novo* biogenesis proceeds along either an anaerobic or aerobic metabolic pathway. The aerobic synthesis involves reduction of the centrally chelated cobalt metal ion of the corrin ring from Co(II) to Co(I) before adenosylation can take place. A corrin reductase (CobR) enzyme has been identified as the likely agent to catalyse this reduction of the metal ion. Herein, we reveal how *Brucella melitensis* CobR binds its coenzyme FAD (flavin dinucleotide) and we also show that the enzyme can bind a corrin substrate consistent with its role in reduction of the cobalt of the corrin ring. Stopped-flow kinetics and EPR reveal a mechanistic asymmetry in CobR dimer that provides a potential link between the two electron reduction by NADH to the single electron reduction of Co(II) to Co(I).

## Abstract 2

CobR is a corrin reductase involved in the biosynthesis of adenosylcobalamin. Using a range of biophysical techniques, we have obtained molecular details on substrate binding and investigated protein stability. Kinetic experiments have identified half-site reactivity.

## INTRODUCTION

Cobalamin, the biologically active form of vitamin B_12_, is an exquisitely complex natural product whose synthesis is limited to only some archaea and bacteria. It functions as a coenzyme or cofactor in a number of important processes, and is particularly associated with isomerization, methylation and dehalogenation reactions [1]. As a modified tetrapyrrole, adenosylcobalamin is constructed along a branched pathway requiring in the region of 30 enzymatic activities for its complete *de novo* synthesis [2]. At the heart of the molecule lies a corrin ring containing a centrally chelated cobalt ion. The cobalt ion is further coordinated by two axial ligands. The lower axial ligand is a dimethylbenzimidazole (DMB) moiety that is attached to the corrin ring through an aminopropanol linker, with the upper axial position occupied by either a methyl or adenosyl group covalently linked to the cobalt. It is the properties of this unique cobalt–carbon bond that facilitate the exceptional chemistry associated with cobalamin-dependent enzymes [1].

The attachment of the upper axial ligand occurs relatively late in the pathway and is catalysed by the ATP:cob(I)alamin adenosyltransferase, which enables the transfer of the adenosyl moiety from ATP to cob(I)yrinic acid a,c-diamide [3]. A prerequisite for this reaction is the 1 electron reduction of the cobalt ion to the Co(I) oxidation state. This reduction is highly challenging and thermodynamically unfavourable, with a midpoint reduction potential of −450 mV for the Co^2+/1+^ coupled reaction [4,5].

The enzyme responsible for the reduction of the cobalt ion was initially purified by Rhone-Poulenc scientists as part of their studies into the molecular genetics of the pathway [6]. However, they failed to identify the gene encoding this enzyme and it was only relatively recently that the enzyme, CobR, was formally characterized in *Brucella melitensis* [7]. The crystal structure of CobR was solved to 1.6 Å resolution and found to be a homodimeric flavoprotein with a similar topology to other characterized flavoproteins such as HpaC and PheA2; however, the CobR dimer was found to have FMN bound to only one sub-unit and this flavin was preferentially crystallized over FAD (flavin dinucleotide; i.e., no additional flavin was added for crystallization). This was considered unusual because FAD was predicted to be the preferred physiological cofactor. In addition, this structural study also demonstrated the transformation of cob(III)alamin into cob(II)alamin in the presence of ATP and NADH was CobR dependent. However, many cobalamin-producing organisms lack a direct orthologue of CobR, especially those that operate an anaerobic biosynthetic pathway. Moreover, it has also been shown that when cobalamin is bound to the adenosyltransferase it is possible to accomplish this reductive step with either a combination of FldA/MSR or with free reduced flavin alone [8,9]. This is facilitated by the way in which cobalamin is bound to the adenosyltransferase, holding the molecule in an unusual 4-coordinate state [5]. Nonetheless, in the aerobic pathway CobR appears to be maintained for the biosynthesis of adenosylcobalamin and a homologue of the protein, PduS, is an integral component of the propanediol utilization machinery, providing compelling evidence that, under some circumstance, these enzymes play an essential role in the adenosylation process [10–12].

Here, we reveal how *B. melitensis* corrin reductase (CobR) binds its coenzyme FAD and also show that the enzyme can bind a corrin substrate. With the coenzyme in excess, a precise two-fold symmetry of the homodimer is demonstrated but under other conditions, important asymmetries are revealed which may help explain how CobR links the two-electron reduction by NADH to the single electron reduction of Co(II) to Co(I).

## MATERIALS AND METHODS

### Recombinant protein production and purification

CobR was overproduced in *Escherichia coli* BL21 Star (DE3) pLysS from a pET14-b construct and purified as described previously [7]. Isotopic labelling for NMR studies was achieved by culturing the *E. coli* strain in M9 minimal media, which was enriched with either ^15^N ammonium sulfate or ^13^C glucose and ^15^N ammonium sulfate (Goss Scientific). Following cleavage of the His-tag with thrombin CobR was further purified by gel filtration. Samples were separated on a Superdex G75 column (GE Healthcare) in 20 mM potassium phosphate buffer (pH 6.5) containing 100 mM NaCl.

### Multidimensional NMR spectroscopy

NMR experimental data for CobR were obtained at 25 and 60°C from 2.0 mM samples, prepared in 20 mM sodium phosphate, 100 mM sodium chloride at pH 6.5. Sample volumes of 330 μl were placed in Shigemi BMS-005V tubes and included 10% D_2_O. All NMR data were acquired at 14.1 T (600 MHz ^1^H) using a Varian UnityINOVA equipped with a 5 mm HCN z-pulse field gradient probe and a 14.1 T (600 MHz ^1^H) Bruker Avance III equipped with a 5 mm QCI-F cryoprobe. Chemical shift referencing was based on the position of the water resonance with the exact value being related to the known relationship of the ^1^H_2_O resonance with temperature [13]. Unless otherwise stated, all NMR experiments were solvent suppressed to reduce the water signal using WATERGATE [14] that was typically obtained using a gradient field strength of 40–50 G cm^−1^. All indirect NMR dimensions were acquired using the hypercomplex method [15] and all reported data numbers reflect the total of real and imaginary points collected in each dimension. All NMR data processing was carried out using NMRPipe [16], chemical shifts were assigned using the CCPN Analysis package [17,18]. Backbone chemical shift assignment of CobR was completed using ^15^N,^1^H-HSQC, CBCANH, CBCA(CO)NH and where necessary ^15^N-edited NOESY, and ^13^C-edited NOESY experiments collected at 60°C. Minimal chemical shift differences are essentially estimated chemical shift perturbations that are obtained using one fully assigned spectrum and measuring the chemical shift distance from signals in the assigned spectrum to the nearest peak in the unassigned spectrum. For ^15^N,^1^H-HSQC datasets, the chemical shift difference is calculated between each pair of peaks using the following expression where Δ^1^H and Δ^15^N are the chemical shift differences in each dimension. Δ^15^N is multiplied by 1/6 to correct for the larger chemical shift range in ^15^N.

(1) $$\mathrm{Shift}\;\mathrm{difference}=\sqrt{{\left(\Delta^{1}H\right)}^{2}+\frac{1}{6}{\left(\Delta^{15}N\right)}^{2}}.$$

NMR relaxation experiments for ^15^N *T*_1_, *T*_2_ and heteronuclear NOE (nuclear Overhauser effect) determination were acquired with the same spectral resolution as HSQC (heteronuclear single-quantum coherence) experiments for CobR under excess FAD conditions at 10 ^15^N *T*_1_ experiments were completed with relaxation delays of 64, 128, 256, 384, 512, 640, 768 and 896 ms including repeats for 250 and 640 ms delays. ^15^N *T*_2_ data were collected with 10 different relaxation delays of 20, 40, 60, 80, 100, 120, 140 and 160 ms including repeats for 80 and 160 ms delays. Heteronuclear NOE experiments were acquired with a transient cycle of 5.5 s both with and without saturation of amide protons. All NMR data were processed using NMRpipe [19] on Linux PC's and all NMR spectra were assigned and relaxation data analysed using the software package CCPN Analysis [17,18]. ModelFree4.0 was used to provide order parameter analysis of ^15^N NMR relaxation data where the chosen models provided optimal chi-squared values for either a one- or two-parameter fit of order parameter only (*S*^2^) or order parameter with additional contributions from chemical exchange (*S*^2^+*R*_ex_).

### Crystallography

Protein crystals were obtained using the hanging drop vapour diffusion method and trays maintained at constant temperature of 18°C. Each trial consisted of a 4 μl drop containing an equal mixture of protein solution (0.4 mM CobR in 20 mM Tris, pH 8.0, containing 100 mM NaCl and 2 mM FAD) and precipitant. Hanging drops were allowed to equilibrate against 1 ml of reservoir solution. Conditions were initially screened using the Molecular Dimensions Structure Screen 1. Following optimization bright yellow crystals were obtained from 0.2 M (NH_4_)_2_SO_4_ and 0.02 M Na acetate, pH 4.6.

Crystals of CobR were transferred into a cryoprotectant solution (0.2 M (NH_4_)_2_SO_4_, 0.02 M Na acetate, pH 4.6 and 20% glycerol) and plunged into liquid nitrogen. Diffraction data were recorded at SRS Daresbury and the crystallographic data are summarized in Supplementary Tables S1 and S2 (at http://www.bioscirep.org/bsr/034/bsr034e120add.htm). Data were processed and reduced using MOSFLM [20] and SCALA [21], solved using MOLREP [22], and refined and visualized using REFMAC5 [23] and COOT [24].

### Fluorescence denaturation studies

Data were obtained using a Varian Cary Eclipse Spectrometer, to provide a measure of conformational stability. The enzyme was incubated for 24 h, to ensure equilibrium had been reached, in Tris buffer (20 mM Tris and 100 mM NaCl, pH 7.5) in the presence of increasing GdnHCl and urea concentrations (0–6 M). All buffers are filter sterilized (0.45 μm filter). The protein concentration used was 30 μg ml^−1^. Fluorescence measurements were obtained from 300 to 450 nm after excitation at 280 nm at 20°C in order to predominantly excite the single tryptophan at position 112. The excitation slit and emission slit were both set at 5 nm. Blanks were run and adjusted for at each concentration. Δ*G* values were obtained from fluorescence at maximum emission using methods described previously [25].

### Differential scanning calorimetry (DSC)

DSC was performed using an automated Microcal capillary DSC instrument (GE Healthcare). Samples were dialysed into 50 mM NaPi, 100 mM NaCl buffer, pH 6.5, and measured at protein concentrations between 50 and 300 μM. Measurements were performed using a standard scan rate of 125°/h with dialysis buffer in the reference cell. Raw data were corrected for the instrumental baseline measured with dialysis buffer in both sample and reference cells and were normalized for protein concentration.

### EPR

EPR spectra were obtained using a Bruker ELEXSYS E500/580 EPR spectrometer operating at X-band. Temperature control was effected using Oxford Instruments ESR900 and ESR935 cryostats interfaced with an ITC503 temperature controller. Samples were prepared as described in the text and then frozen in liquid nitrogen. Experimental conditions were as given in the figure caption.

### Stopped-flow kinetic experiments

Stopped-flow kinetic analysis of the reduction of CobR was performed under anaerobic conditions in a glove box (Bell Technology), maintained at less than 2 ppm oxygen, with an Applied Photophysics SX20 stopped-flow spectrometer. All experiments were carried out in single mixing mode and monitored by multiple wavelength diode array or single wavelength analyses at 455 nm. CobR (5 μM) was rapidly mixed with NADH (10–250 μM) (final concentration after mixing) in 20 mM Tris, pH 8.0, containing 100 mM NaCl at 25°C. Single wavelength traces at 455 nm were best described by a double exponential function using the following equation:

(2) $$A=A_{1}\exp\left(-k_{1\mathrm{obs}}t\right)+A_{2}\exp\left(-k_{2\mathrm{obs}}t\right)+C.$$

where *k*_1obs_ and *k*_2obs_ are the observed rate constants for the two phases, *A* is the absorbance at time *t*, *A*_1_ and *A*_2_ are the amplitudes for each phase and *C* is the residual absorbance at the end of the reaction. The data for the concentration dependence of NADH on *k*_1obs_ were assumed to follow a three-step model as indicated below:

$$E_{\mathrm{ox}}+\mathrm{NADH}\overset{K_{1}}{\Leftrightarrow }E_{\mathrm{ox}}\mathrm{NADH}\overset{k_{2_{}}}{\underset{k_{-2}}{\Leftrightarrow }}E_{\mathrm{ox}}^{t}\mathrm{NADH}\overset{k_{3_{}}}{\underset{\Delta \mathrm{abs}}{\to }}E_{\mathrm{red}}{\mathrm{NAD}}^{+}$$

The *k*_1obs_ data were fitted to the following hyperbolic equation:

(3) $$k_{1\mathrm{obs}}=\frac{k_{1}k_{2}\left[\mathrm{NADH}\right]}{k_{1}\left[\mathrm{NADH}\right]+1}+k_{-2}$$

where *K*_1_ is the apparent affinity for NADH and, since *k*_−2_≈0, *k*_2_ represents the maximal rate of flavin reduction (*k*_max_).

## RESULTS

### Chemical shift assignments

**Figure 1:**
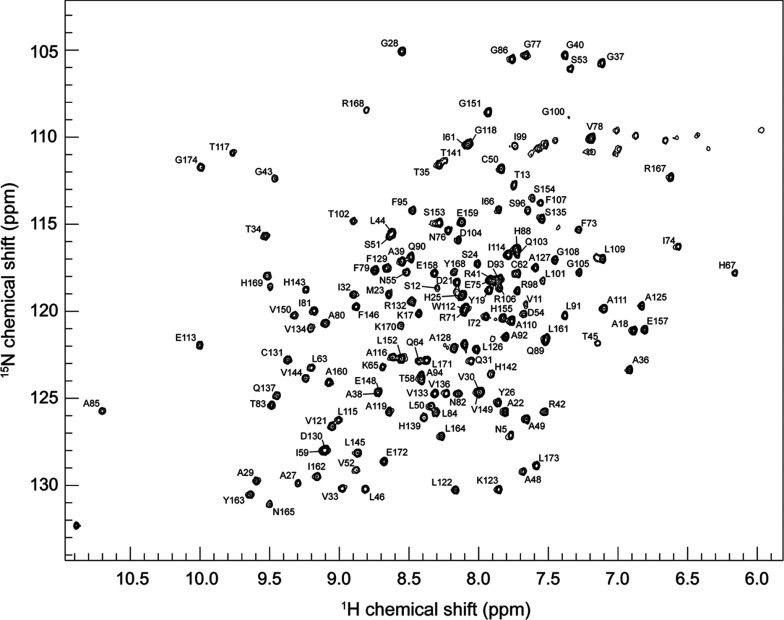
^15^N,^1^H-15HSQC spectra of *B. melitensis* CobR at 60°C Assignments for the backbone cross peaks are shown using one-letter amino acid code and sequence number.

Backbone chemical shift assignments were completed for *B. melitensis* CobR with 94%, 94% and 86% assignments, respectively, for backbone NH, C_α_ and C_β_ resonances. The assigned backbone amide resonances of the ^15^N,^1^H-HSQC spectrum are shown in Figure 1. The definitions and limits of the NMR solution-based secondary structure, in comparison with those observed for the protein crystal structure 3CB0.pdb [7], were confirmed by means of a DANGLE analysis [26] (Supplementary Figure S1 at http://www.bioscirep.org/bsr/034/bsr034e120add.htm). The uncomplicated nature of Figure 1 supports the existence of a single species for CobR when in solution. The protein was confirmed as being dimeric in solution by gel filtration chromatography at room temperature (Supplementary Figure S2 at http://www.bioscirep.org/bsr/034/bsr034e120add.htm) and by subsequent NMR relaxation measurements at 60°C.

### ^15^N NMR relaxation analysis of CobR

**Figure 2:**
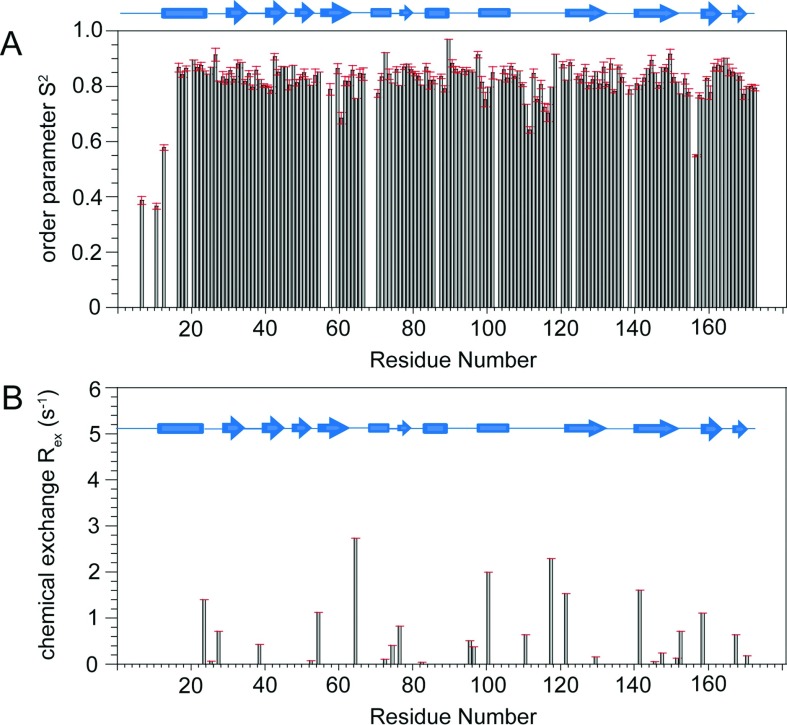
The variation of order parameter *S*^2^ (A) and chemical exchange contribution R_ex_ (B) following ModelFree analysis of ^15^N NMR relaxation data for *B. melitensis* CobR at 60°C The secondary structure of *B. melitensis* CobR is shown across each plot.

^15^N *T*_1_, *T*_2_ and heteronuclear NOE NMR data for CobR (Supplementary Figure S3 at http://www.bioscirep.org/bsr/034/bsr034e120add.htm) were analysed using ModelFree 4.0 with results shown in Figure 2 using a global correlation time *τ*_m_ of 9.44±0.93 ns that was estimated from the average *T*_1_/*T*_2_ ratio over the structured regions. A total of 146 residues was able to provide ModelFree data of which 26 required a two-parameter fit to *S*^2^+*R*_ex_ with the remaining 120 requiring only a single parameter fit to *S*^2^. Over the structured region of CobR, the average *S*^2^=0.84 with a standard deviation of 0.04. All *R*_ex_ contributions were modest with an average of 0.77 s^−1^ across all 26 residues with no specific identifiable pattern to structural elements observed.

### Crystallization of CobR with excess flavin

**Figure 3:**
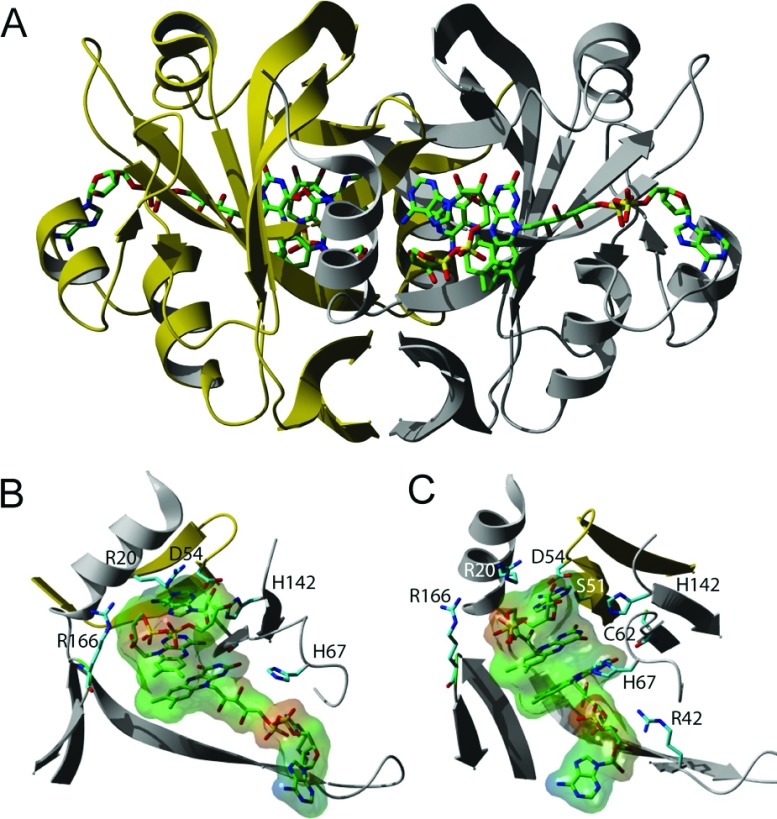
Structure of CobR showing multiple occupancy by FAD Ribbon diagram of the crystal structure of *B. melitensis* CobR with two molecules of FAD (green sticks) bound to each monomeric unit in gold and grey (**A**). Close up of the binding site highlighting key amino acids that interact with FAD (**B**). (**C**) is identical with (**B**) but rotated through 45° about the *z*-axis. FAD in (**B**) and (**C**) is shown complete with a molecular surface.

The original crystal structure of CobR (PDB code: 3CB0) had only one bound FMN nucleotide per dimer, while all other biochemical evidence suggested that there should be two molecules of FAD bound per dimer [7]. The bright yellow crystals of CobR grown here were in the presence of excess FAD and afforded a structure at a maximum resolution of 2.2 Å, which was solved by molecular replacement using 3CB0 as the search model. The final crystallographic model (Figure 3) contains 162 amino acid residues with an *R*_work_ of 22.4% and an *R*_free_ of 25.0% (Supplementary Table S2). Unlike the previous structure, FAD is seen occupying both active sites of the homodimer. The CobR FAD binding site comprises residues from both subunits (Figure 3b) and highlights the importance of the CobR homodimer in binding FAD. Interactions between Ser-51 and Asp-54 from one subunit are combined with interactions between Arg-20, Arg-42, Cys-62, His-67, His-142 and Arg-166 from the second subunit for the flavin bound in each site.

The peptide backbone of the structure with FAD bound is essentially identical with the FMN bound structure with an r.m.s.d. of 0.99 Å over 162 equivalent C_α_ atoms. This demonstrates that the binding of FAD has no effect on the tertiary structure of the protein and that no movement of the backbone is necessary to accommodate the addition of the AMP moiety of FAD compared with FMN.

From the analysis of the *F*_0_−*F*_c_ electron density map it was possible to identify 4 molecules of FAD bound per dimer. The isoalloxazine and ribotyl moieties of the first FAD molecule can be superimposed with the FMN in the FMN bound structure with only minor deviation and the same protein contacts are present and remain unchanged. However, the AMP moiety of FAD is not bound in the same manner as any of the other members of this flavoprotein family. CobR is an unusual member of the family since, like TftC [27], it possesses an extra helix (α4) which resides in the region of the binding site for the AMP group (Supplementary Figure S4 at http://www.bioscirep.org/bsr/034/bsr034e120add.htm). The binding modes observed in the structures of PheA2 [28] and HpaC [29] are precluded due to the presence of this aforementioned helix. In the CobR/FAD structure, the adenine group is packed against the loop between helix α3 and helix α4 and is primarily in contact with Leu-101 and Thr-102 while the ribose ring is anchored in place with interactions through Gln-103.

The additional FAD molecule in each subunit is located in the active site cavity and adopts a folded conformation similar to that observed for the binding of NADH to other flavin reductases with the adenine group stacked against the isoalloxazine ring system. The isoalloxazine moieties of both flavins are also in close proximity with an interflavin N5–N5 distance of 3.8 Å. The folded FAD is hydrogen bonded to the side chains of His-142, Arg-166 together with Ser-51 and Asp-54 from the opposite polypeptide chain across the dimer interface (Supplementary Figure S5 at http://www.bioscirep.org/bsr/034/bsr034e120add.htm).

### Addition of excess FAD

**Figure 4:**
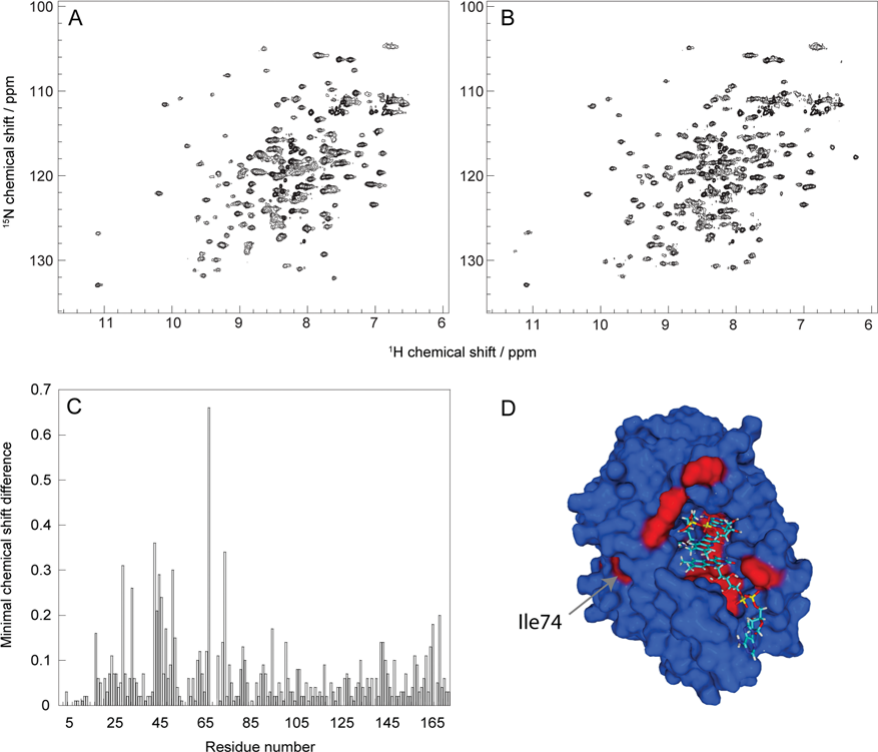
Chemical shift mapping of FAD binding to CobR ^15^N,^1^H-HSQC NMR spectra of CobR at 25°C as purified and concentrated (**A**) and with excess FAD (**B**). ^15^N,^1^H minimal chemical shift difference using assignments for CobR with excess FAD is shown (**C**) together with a surface image of CobR with excess FAD (**D**) where residues with minimal map values >0.15 are shown in red and highlighting Ile-74. Multiple bound FAD molecules are shown as sticks in (**D**).

^15^N,^1^H-HSQC spectra of CobR in the absence of excess FAD (Figure 4a) illustrate that CobR is folded, structurally stable and symmetrical. However, significant changes in the NMR data are observed on addition of excess FAD (Figure 4b), which can be mapped to specific residues (Figure 4c). Changes in the chemical shift map, greater than the standard deviation from the mean minimal chemical shift difference, are observed for residues adjacent to the flavin binding site: Lys-17, Lys-29, Val-33, Gly-43, Leu-44, Thr-45, Leu-46, Ala-48, Ser-51, Val-52, His-67, Phe-95 and Arg-166. Ile-74 and His-169 are highlighted as possessing significant chemical shift changes upon addition of excess FAD but are not residues adjacent to the flavin binding site. This suggests that a small but significant degree of conformational movement occurs in CobR upon binding an additional 2 molecules of FAD. Ile-74 is highlighted in Figure 4(d) to the left of the binding site and His-169 (in an equivalent position to Ile-74 on the opposite side of the homodimer) also displays a significant chemical shift change upon FAD binding.

### Chemical denaturation studies

The chemical stability of CobR was assessed by following the unfolding of the protein using intrinsic fluorescence; as denaturant concentration was increased unfolding causes the buried tryptophan inside CobR to become exposed. Fluorescence intensity is at a maximum and at a shorter wavelength of 320 nm when CobR is folded which shifts to a maximum emission at a longer wavelength of 350 nm when the protein unfolds. To assess fully the chemical denaturation, CobR was unfolded in the presence of two denaturants: urea and guanidinium hydrochloride.

**Figure 5:**
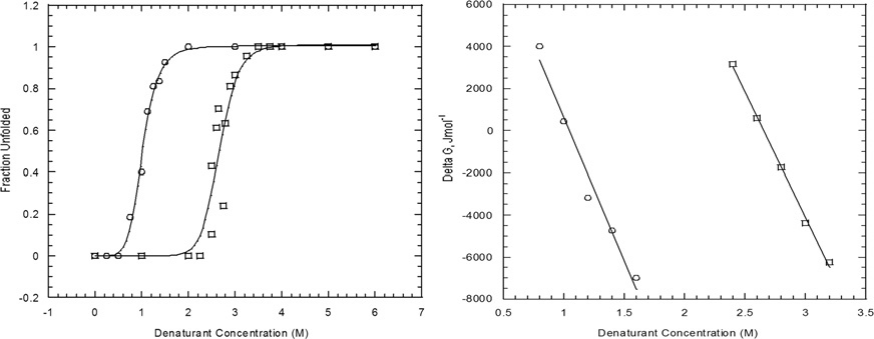
Protein denaturation curves for CobR Fraction folded and unfolded from intrinsic fluorescence denaturation of *B. melitensis* CobR in (□)-urea, (○)-GdnHCl including a Hill equation curve fit (**A**). Variation in Δ*G* with denaturant concentration to ascertain Δ*G* at zero denaturant concentration (**B**).

Both unfolding curves can be fitted using a two-state approximation with no folding intermediates detected (Figure 5). Fluorescence data collected revealed Δ*G* for urea denaturation of 31.6±1.6 and 14.2±0.004 kJ mol^−1^ for GdnHCl. Errors here reflect the precision of fit to a two-state approximation. A variation in Δ*G* is seen due to the different denaturant properties. GdnHCl interacts via electrostatic interactions due to its ionic properties; however, urea operates through chaotrophic effects [30]. This suggests that electrostatic contributions are important for CobR stability.

### DSC

The melting point (*T*_m_) of *B. melitensis* CobR was determined to be 70°C by DSC; this value is assumed and not actual as the unfolding reaction is not reversible. An increase in heat capacity was observed in each rescan due to the exposure of hydrophobic residues to water and the melting temperature was also seen to depend on the scan rate. The apparent *T*_m_ increases with the scan rate due to kinetic effects that drives the equilibrium from dimer to subunit to aggregate. At higher scan rates the protein appears more thermostable as the equilibrium between the unfolding of dimer to subunit and then to form aggregates is not complete. It was also shown that excess FAD has an effect on apparent *T*_m_ which shifted to a value of 80°C; a property usually expected for a non-covalent ligand.

### Cobinamide binding

**Figure 6:**
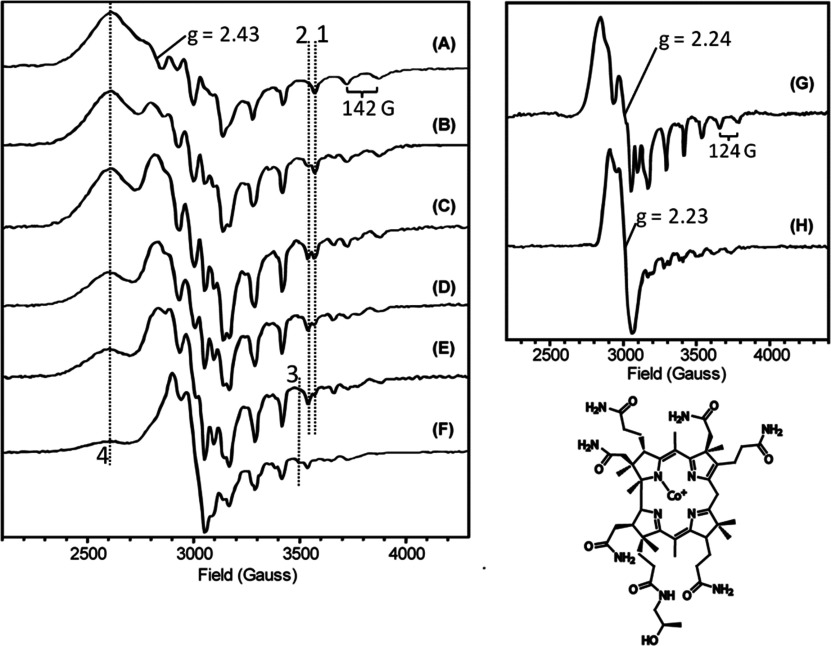
X-band EPR spectra showing the titration of Co^2+^-cobinamide with CobR (**A**) 250 μM Co^2+^-cobinamide, (**B**) 0.25 equivalent of CobR, (**C**) 0.5 equivalent of CobR, (**D**) 1 equivalent of CobR, (**E**) 1.5 equivalents of CobR and (**F**) 2 equivalents of CobR. At two equivalents of CobR, a second base-on form of cobinamide is observed with *g*_⊥_=2.23. (**G**) The resulting spectrum after the subtraction of the base-off component from spectrum D and (**H**) spectrum of the second base-on species generated by the subtraction spectra A and G from spectrum F. Protein equivalents are expressed per dimer. Spectra were recorded at 20 K employing a microwave power of 500 μW, modulation frequency of 100 kHz and modulation amplitude of 5 G. Also shown is the molecular structure of cobinamide. See text for further explanation.

The binding of a corrin substrate to CobR was investigated by EPR. The addition of CobR to a solution of Co^2+^-cobinamide revealed that the coordination of the cobalt ion changed from a 5-coordinate, low spin species with a water axial ligand, with *g*_∥_=2.43 and *A*_∥_=142 G (equivalent to a base-off cobalamin spectra), to a mixture of species with differing axial ligation, with *g*_∥_=2.24 and *A*_∥_=124 G (Figure 6). The observed decrease in the hyperfine coupling (*A*_∥_) following the addition of CobR signifies a decrease in the unpaired spin density on the cobalt centre, which can be attributed to strengthening of the cobalt axial ligand bonding interaction [31]. The conversion from the water axial ligand to the newly identified species can be followed by observing the *g***_∥_** hyperfine lines marked 1 and 2. In Figure 6(C) these two are of approximately equal intensity, indicating that the conversion is 50% complete at half an equivalent of CobR.

At a ratio of one cobinamide per CobR (i.e., 2 per dimer) (Figure 6D), the spectrum was a mixture of the two forms of cobinamide and subtraction of the initial water ligated component gives the spectrum of the new species (Figure 6G). No superhyperfine splitting is observed in the resulting spectrum which suggests that the fifth ligand does not use nitrogen or the ligand is not orientated directly along the *z*-axis. However, at two equivalents of CobR (i.e., 1 per dimer), a third form of cobinamide, with *g*_∥_=2.23, becomes prominent (Figure 6F, see also the new *A***_∥_** hyperfine feature marked 3). Subtraction of both the water-ligated component and the species observed in Figure 6(G) generates a new spectrum, which shows a superhyperfine splitting of the *g*_∥_ region (18 G) indicating coupling to a nitrogen (^14^N) nucleus (Figure 6H). This is thought to arise from the axial ligand being formed by a CobR side chain, e.g. a histidine imidazole group. Note that intensity at the position marked 4 shows that the water-ligated form persists to Figure 6(F).

The binding of Co^2+^-cobinamide to CobR was also investigated by NMR. The addition of cobinamide to a sample of CobR-induced chemical shift changes which were observed in the ^15^N,^1^H-HSQC and also the HNCO spectra. When the key differences were mapped on to the structure of CobR it was evident that the changes are not localized to any particular region of the protein and are in fact more indicative of a global change in the structure (Supplementary Figure S6 at http://www.bioscirep.org/bsr/034/bsr034e120add.htm).

### Reduction of CobR by NADH

**Figure 7:**
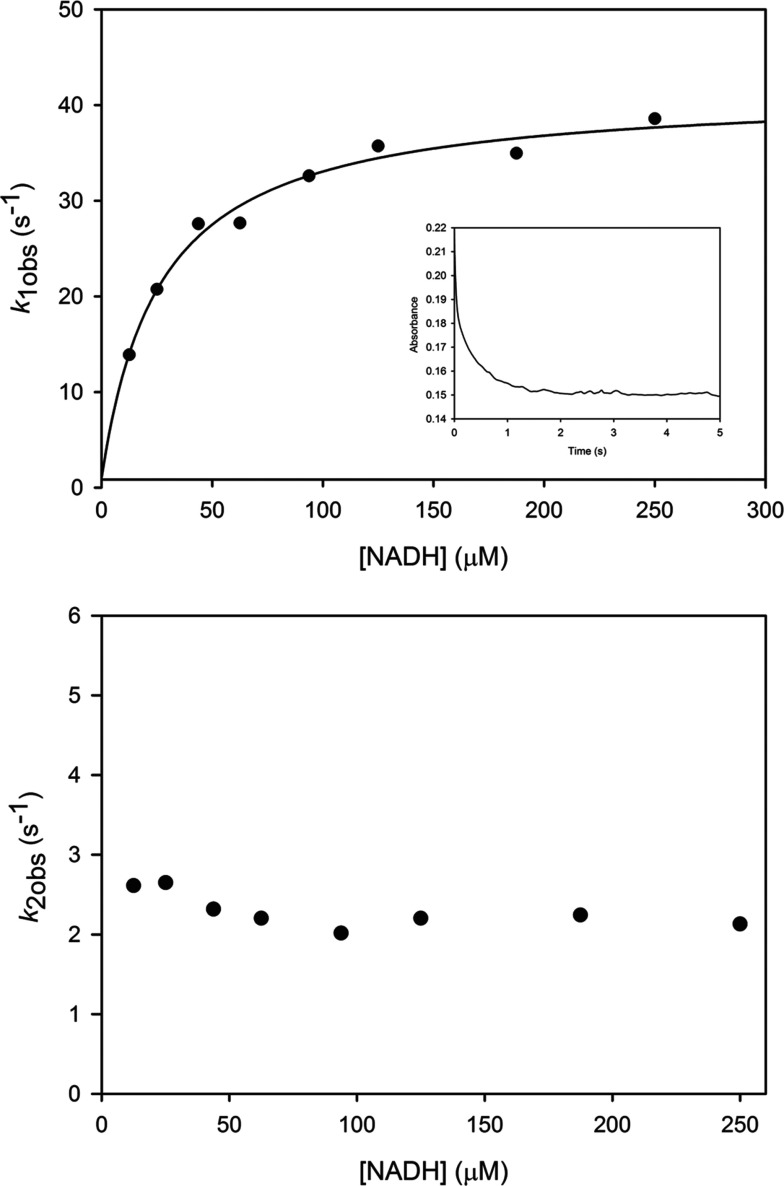
The dependence of the observed rates of CobR reduction on NADH concentration Observed rate constants were obtained by fitting absorbance transients collected at 455 nm following the rapid mixing of CobR (5 μM) with NADH (10–250 μM) under anaerobic conditions at 25°C to a double exponential function revealing *k*_1obs_ and *k*_2obs_. The data for *k*_1obs_ were fit to a hyperbolic function yielding a *K*_1_ of 24.8±2.6 μM and a maximal rate of reduction *k*_max_ of 41.3±1.1 s^−1^. Inset is the absorbance transient recorded at 455 nm upon mixing 5 μM CobR with 125 μM NADH.

The reaction mechanism of CobR involves the reduction of the bound flavin prosthetic group by NADH. This reductive half reaction was investigated under anaerobic conditions using the stopped-flow apparatus. Absorption transients at 455 nm were recorded following the mixing of CobR (5 μM) with NADH (10–250 μM) at 25°C. These time-dependent data were best fitted to a double exponential function representing two distinct phases of the reaction. The first phase (*k*_obs1_) shows a rapid initial decrease in the absorbance of the oxidized flavin and a hyperbolic dependence on NADH concentration. The second phase (*k*_obs2_) was slower and there was no observable dependence on nucleotide concentration (Figure 7). The two phases carry equal amplitude and thus are each responsible for 50% of the flavin absorbance change during reduction. This is a clear indication of half site reactivity, where the two phases represent the reduction of the flavin at each of the active sites in the dimer. No kinetic isotope effects were observed for either of the phases when following the reduction of CobR by either [(4*R*)-^2^H)]NADD or [(4*S*)-^2^H)]NADD. The observed rate constant for the first phase (*k*_obs1_) against NADH concentration was fitted to a hyperbolic function (eqn 3) yielding an apparent affinity (*K*_1_) of 24.8±2.6 μM and a limiting maximal rate of reduction (*k*_max_) of 41.3±1.1 s^−1^. This suggests that following the rapid binding of NADH to CobR, a rearrangement or conformational change is required before hydride transfer from the pyridine nucleotide. This conformational change is the rate limiting step in the reduction of the flavin and precedes a faster hydride transfer reaction. The reduction of the first flavin in the dimer, together with the associated change in conformation, greatly affects the rate of reduction at the second site. This implies communication across the dimer interface and half site reactivity.

## DISCUSSION

NMR analysis of CobR through both chemical shift assignments and DANGLE analysis reveals that the solution conformation of the protein is comparable with the crystal structure and verifies the use of NMR as an additional structural probe to interrogate the CobR. The well-resolved ^15^N HSQC are consistent with a symmetrical CobR homodimer. If the dimer was asymmetric, the NMR data would reflect the structural non-equivalence of each subunit and would manifest in the HSQC as NMR resonance doubling. It is possible that two non-equivalent units could interconvert providing a single average spectrum but ^15^N NMR relaxation data in Figure 2 and Figure S3 do not reveal any abnormally low spin-spin relaxation rates that would indicate exchange line broadening. Equally, ModelFree analysis confirms that CobR behaves as a compact species in solution with an average order parameter *S*^2^ of 0.84. The limits of *S*^2^ define a completely rigid model at *S*^2^=1.0 and randomly flexible model at *S*^2^=0.0 with a value of 0.84 being within the typical range for a globular protein species. In addition, the estimated correlation time of CobR, from ^15^N relaxation data, supports the presence of a 36 kDa homodimer in solution at 60°C; estimating *τ*_m_ at 60°C for a 174 and 348 residue globular protein [32] provides 4.64 and 8.84 ns, respectively. The estimated *τ*_m_ of 9.44±0.93 ns clearly supports the dimer species being present in solution. The relaxation parameters show faster motion with increased *T*_1_ times at two loops, between β4 and α2 and α4 and β6. The first loop contains residues Gln-64 and His-67, known to hydrogen bond to the isoalloxazine ring [8]. In the FMN bound structure, helix α4 is present in the adenine-binding pocket, revealing that movement of this helix and flexibility of the preceding loop allows binding of FAD. This is confirmed by the ^15^N-^1^H HSQC spectra in the presence and absence of FAD which show significant chemical shift differences at amino acid residues within the loop (Figure 4).

The latest crystal structure of CobR under conditions of excess FAD has confirmed that both active sites are able to bind flavin. At this elevated concentration of flavin, each active site was occupied by two flavin molecules which were pi–pi stacked. This crystal structure highlights the similarities of CobR to flavin reductases PheA2 and TftC. Each binding site in these flavin reductases contain one FAD molecule and is able to bind an additional flavin or NADH molecule. Crystal structures of PheA2 and TftC have been obtained with both the flavin prosthetic group and the nicotinamide cofactor bound in the active site [27,28]. When comparing these structures with CobR, it can be clearly seen that the additional FAD molecule in CobR is configured in a curled fashion that is extremely similar to the conformation of FAD/NADH in PheA2 and TftC as highlighted in Supplementary Figure S4.

Stability studies reveal CobR to be a thermostable protein with a melting temperature of 70°C, which is enhanced in the presence of the cofactor FAD to 80°C. Cofactors can aid protein stability, of course, through preferential binding to the native state [33,34]. Also, from Le Chatelier's principle and the laws of mass action when FAD is in excess, it becomes energetically unfavourable to displace the cofactor from the protein into the solution. Therefore, when both FAD binding sites on each CobR dimer are saturated, excess FAD will stabilize the protein further due to entropy effects. Interestingly, a flavin reductase from the mesophilic organism *Sinorhizobium meliloti* has also been identified with 43% sequence identity, and NMR data confirm a conserved structure to the *B. melitensis* CobR (results not shown). DSC studies of the flavin reductase from *S. meliloti* revealed an apparent *T*_m_ of 72°C (results not shown), which raises the question of whether enhanced thermostability or structural rigidity is an evolutionary adaption that is required for the function of the CobR enzyme.

Despite the protein exhibiting thermal stable properties the chemical denaturation study confirmed the protein can be unfolded at relatively low concentrations of denaturant. Unfolding curves show a two-state folding mechanism, once unfolding occurs to the dimer it precedes to an unfolded state. Differences between the chemical stability estimations via urea and GdnHCl are widespread through the literature. For example, Human Placental Cystatin, a thiol proteinase inhibitor, has a midpoint of transition of 1.5 M in GdnHCl and 3 M in urea [35]. A complete understanding of the mechanism of unfolding by urea and GdnHCl is not known due to the complexity of folded proteins; however, it has been proposed that the difference between the two values can be attributed to electrostatic interactions. There are six salt bridges that can be identified from the structure of CobR: (Lys-17/Asp-21), (Arg-20/Asp-54), (Lys-65/Asp-138), (Arg-71/Glu-75), (Arg-98/Asp-93) and (Arg-132/Glu-148). These salt bridges have the potential to form the basis of the thermostability observed; a hypothesis supported by ΔΔ*G* of denaturation (Δ*G*_urea_−Δ*G*_GdnHCl_) of 17.4 kJ mol^−1^ supporting a significant electrostatic contribution to conformational stability.

It has been suggested that for an enzyme to be designated a CobR there should be evidence not only for reduction of the cobalt but also corrin binding [8]. Herein, we have presented evidence from both NMR and EPR studies that CobR is able to bind cobinamide. The binding of a corrin to CobR induces global electronic changes within the protein, which alter the properties of both active sites across the dimer.

Interestingly, CobR seems to provide an axial ligand for the cobalt ion. This is similar to what is observed with the binding of cobalamin to PduS [11], the cobalt reductase associated with 1,2-propanediol metabolism. Furthermore, both NMR and the crystallographic structure of CobR in excess FAD support the symmetrical multiple flavin occupancy of the active site in a manner similar to PheA2 and TftC that utilize hydride transfer with NADH. This suggests parallels in the mechanism in CobR to these flavin reductases where, in the cell, CobR has the potential to exist with each binding site occupied by a flavin and an NADH molecule.

The site of corrin reduction *in vivo* is currently unknown. It is possible that CobR transfers electrons to the adenosyltransferase or alternatively the corrin is reduced while bound to CobR and then passed on the adenosyltransferase enzyme. The fact that CobR has been shown to bind and reduce a range of corrins to the Co^1+^ state even in the absence of an adenosyl transferase [7] perhaps favours the latter. The binding of a Co^2+^-corrin to the adenosyltransferase enzyme appears to induce a 4-coordinate conformation which reduces the thermodynamic barrier for reduction. In this state, a number of flavoproteins and even free reduced flavin have been shown to catalyse the reduction of the corrin [8]. Adenosyltransferase enzymes fall within three unrelated families, CobA, PduO and EutT [36], and the rate of reduction of cobalamin by free flavin varies by the type of adenosyltransferase. With the CobA type of enzyme, which is the form present in *B. melitensis*, the rate of reduction was significantly slower than the PduO type [8]. This may account for why some bacteria require a dedicated reductase. Further investigation is required to explore potential interactions between CobR and the adenosyltransferase.

Reduction of the FAD coenzyme by NADH appears to be at least a three-step process, where a conformational change is required before hydride transfer. The protein displays half site reactivity, where reduction of the flavin in one active site affects the rate of reduction at the second active site in the dimer. This observed conformation change associated with the binding of NADH at one active site is similar to the phenomenon that was observed in the EPR studies looking at cobinamide binding, where two different coordination states were detected depending on the whether one or both of the active sites in the dimer were occupied.

The functional importance and structural basis for the observed half site reactivity are unclear. The two available CobR structures (with either 1 or 4 molecules of flavin bound per homodimer) are essentially identical in structure. Comparison of the structures of Phea2 and TftC with and without NAD similarly also shows very little structural change. Consequently, any structural or dynamic changes occurring upon substrate binding or flavin reduction are subtle. Clearly, the binding of flavin or substrate to one active centre will affect the electrostatic potential at the other. Additionally, subtle changes in dynamics at the second active centre may accompany binding at the first. The two active sites of the CobR homodimer are connected through a water containing channel and it is possible that communication is mediated through this channel.

The half site reactivity will result in one subunit reacting at a time. This may be beneficial to the catalytic process, since the enzyme links the one electron reduction of the corrin with the two electrons obtained from NADH. The fate of the second electron is currently unknown; however, it is known to disproportionate very quickly and this could be intramolecular rather than an intermolecular process. It is also possible that NADH binds to one active site while the corrin substrate docks at the active site on the opposite side of the dimer. Such a process could allow for regulation of the enzyme thereby controlling adenosylcobalamin synthesis. In this respect the functional importance of this half site reactivity requires further investigation.

## Online data

Supplementary data
