# Supplementary material for: FAD binding, cobinamide binding and active site communication in the corrin reductase (CobR)
Source: Biosci Rep. 2014 Jul 4;34(4):e00120. doi: 10.1042/BSR20140060 (PMC4083273; doi:10.1042/BSR20140060)
Supplement: Supplementary data [file bsr034e120add.pdf]

Biosci. Rep. (2014) / **34** / art:e00120 / doi 10.1042/BSR20140060

OPEN ACCESS

Original Paper

Bioscience Reports

www.bioscirep.org

Bioscience Reports

# SUPPLEMENTARY DATA

# FAD binding, cobinamide binding and active site communication in the corrin reductase (CobR)

Andrew D. LAWRENCE*, Samantha L. TAYLOR*, Alan SCOTT*, Michelle L. ROWE*, Christopher M. JOHNSON†, Stephen E. J. RIGBY‡, Michael A. GEEVES*, Richard W. PICKERSGILL§, Mark J. HOWARD*[1] and Martin J. WARREN*[1]

*School of Biosciences, University of Kent, Canterbury, Kent CT2 7NJ, U.K.
†Medical Research Council Laboratory for Molecular Biology, Hills Road, Cambridge CB2 0QH, U.K.
‡Manchester Institute of Biotechnology, University of Manchester, 131 Princess Street, Manchester M1 7DN, U.K.
§School of Biological and Chemical Sciences, Queen Mary, University of London, Mile End Road, London E1 4NS, U.K.

```
1      MQTVNNIISSVSTVESKAYRDAMSHYAGAVQIVTTAGAAGRRGLTLTAACSV
1      MQTVNNIISSVSTVESKAYRDAMSHYAGAVQIVTTAGAAGRRGLTLTAACSV

53     SDNPPTILICLQKIHEENRIFIENGVFAINTLAGPHQQLADAFSGRIGLTQDERFE
53     SDNPPTILICLQKIHEENRIFIENGVFAINTLAGPHQQLADAFSGRIGLTQDERFE

109    LAAWEILATGAPVLKGALAAFDCRVVSVQDHSTHHVLFGEVVGLSSHAEEEA
109    LAAWEILATGAPVLKGALAAFDCRVVSVQDHSTHHVLFGEVVGLSSHAEEEA

162    LIYLNRRYHKLEL
162    LIYLNRRYHKLEL
```

**Figure S1 Secondary structure analysis of CobR from the 3CB0.pdb crystal structure (1st sequence) and NMR data (2nd sequence) using DANGLE**
Alpha helices are shown in yellow and beta-stands shown in green.

[1] Correspondence may be addressed to either of these authors (email m.j.howard@kent.ac.uk or m.j.warren@kent.ac.uk).
The structural co-ordinates reported will appear in the PDB under accession code 4IRA. NMR assignments are available from the BioMagResBank with accession number 16780.

© 2014 The Author(s) This is an Open Access article distributed under the terms of the Creative Commons Attribution Licence (CC-BY) (http://creativecommons.org/licenses/by/3.0/) which permits unrestricted use, distribution and reproduction in any medium, provided the original work is properly cited.

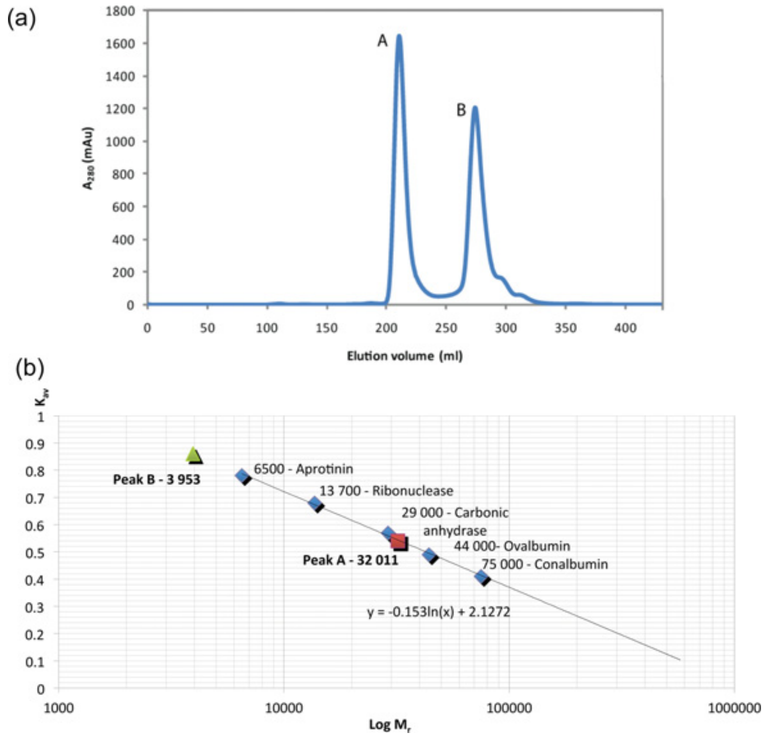


**Figure S2** **CobR characterized by gel filtration chromatography**
The sample was run on a Superdex 200 gel filtration column using a 20 mM sodium phosphate buffer (pH 7.5) containing 100 mM NaCl and excess flavin (**a**). SDS/PAGE analysis confirmed the presence of CobR in the elution fractions. Fractions corresponding to the CobR dimer are seen, peak A at 32.01 kDa, and the FAD cofactor, peak B as shown on the gel filtration calibration curve (**b**).

© 2014 The Author(s) This is an Open Access article distributed under the terms of the Creative Commons Attribution Licence (CC-BY) (http://creativecommons.org/licenses/by/3.0/) which permits unrestricted use, distribution and reproduction in any medium, provided the original work is properly cited.

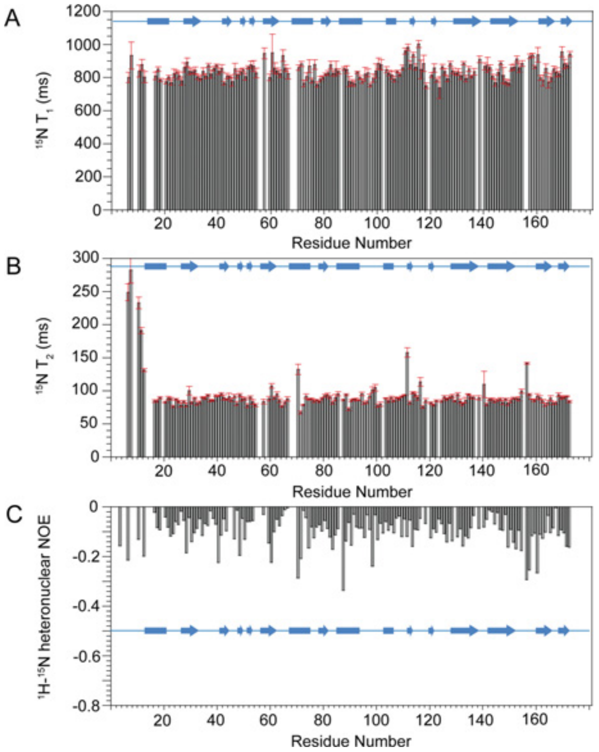


**Figure S3 $^{15}$N NMR relaxation parameters $T_1$, $T_2$ and heteronuclear NOE for CobR at 60°C**
The secondary structure of *Brucella melitensis* CobR is shown across each plot.

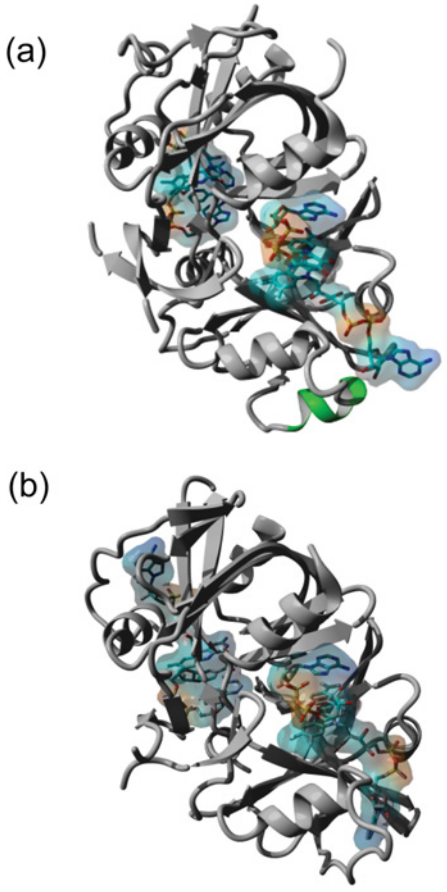


**Figure S4 Ribbon diagram of the crystal structures of *Burkholderia cepacia* TftC (a) and *Geobacillus thermoglucosidasius* PheA2 (b) showing FAD and NAD units as sticks and molecular surfaces to highlight the similar arrangement to FAD binding observed in *Brucella melitensis* CobR**
The additional helix found in TftC and CobR is highlighted in green in (**a**).

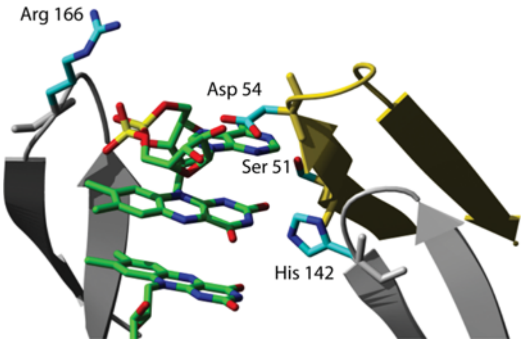


**Figure S5 Structural detail of CobR amino acid side chains (in blue) involved in hydrogen bonding with FAD (green) using gold/grey monomer colours as in Figure 3**

© 2014 The Author(s) This is an Open Access article distributed under the terms of the Creative Commons Attribution Licence (CC-BY) (http://creativecommons.org/licenses/by/3.0/) which permits unrestricted use, distribution and reproduction in any medium, provided the original work is properly cited.

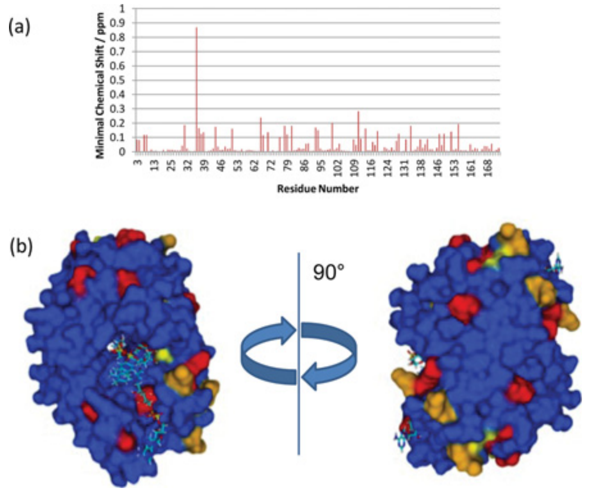


**Figure S6 HNCO minimal chemical shift map for CobR±$B_{12}$ (a) and key shift changes >1$\sigma$ (red) and 0.5$\sigma$ (orange) mapped on the CobR crystal structure**

**Table S1 Crystallographic data collection statistics**

| **Parameter** | |
|---|---|
| Wavelength (λ) | 1.1170 Å |
| Space group | *P*6222 |
| Resolution | 2.2 Å |
| $R_{merge}$ | 0.115 (0.531)* |
| Mean $I/\sigma(I)$ | 17.8 (4.1) |
| Unique reflections | 21883 (3114) |
| % Completeness | 99.9 (100.0) |
| Multiplicity | 10.4 (10.1) |

*The overall resolution range is 53.37–2.20 Å and in parenthesis 2.26–2.20 Å.

**Table S2 Details of the final model refinement**

| **Parameter** | |
|---|---|
| *R* factor | 22.4% |
| $R_{free}$ | 25.0% |
| RMS bond lengths (Å) | 0.027 (0.021)* |
| RMS bond angle (°) | 2.173 (2.060) |
| RMS chiral (Å$^3$) | 0.118 (0.200) |
| Ramachandran plot (%) allowed | 98.09 %† |
| *B*-factors (Å$^2$)‡ | |
| Protein | 19.2 |
| FADs | 22.1 |
| Waters | 31.9 |

*RMS is the root mean square value. The value in the parenthesis is the target value.
†Leu-101, Leu-115 and Ser-140 have clear electron density but fall outside of the allowed region of the Ramachandran plot where they account for the disallowed 1.91 %.
‡The mean *B*-factor from the Wilson plot was 21.8.

**Received 14 April 2014/22 May 2014; accepted 28 May 2014**

**Published as Immediate Publication 9 June 2014, doi 10.1042/BSR20140060**

© 2014 The Author(s) This is an Open Access article distributed under the terms of the Creative Commons Attribution Licence (CC-BY) (http://creativecommons.org/licenses/by/3.0/) which permits unrestricted use, distribution and reproduction in any medium, provided the original work is properly cited.
